# Pathological and Histological Researches on Inflammation of the Nervous Centres

**Published:** 1844-04

**Authors:** 


Art. VII.—*Pathological and Histological Researches on Inflammation of the Nervous Centres.* By John Hughes Bennett, m.d. &c. &c. —*Edinburgh,* 1843. 8vo, pp. 84.

Although this pamphlet comes before the profession as a distinct publication, it is neither a second edition nor a reprint of the series of articles published by the author in the Edinburgh Medical and Surgical Journal, but the articles themselves in a collected form, and printed from the types already set up, with the addition of a title-page. We think it necessary to state so much, for the benefit of those of our readers who have access to the volumes of our contemporary.

These articles consist of a series of cases of cerebral and spinal disease, with a detail of the morbid changes, as they appeared to the eye with and without the microscope; plates exhibiting the microscopic changes are given.

The essay is very creditable to Dr. Bennett, whose principal conclusions are the following: Two kinds of cerebral and spinal softening exist, an inflammatory and a non-inflammatory, which may always be distinguished from each other by means of the microscope; the former being characterized by the presence of exudation-corpuscles, and granules, and essentially consisting in the formation and development of nucleated cells in the liquor sanguinis effused from the vessels and acting as a *blastema.* As these cells become developed, they burst, and render the exudation soft, pultaceous, or even diffluent. On the other hand, non-inflammatory softening is the result of a mechanical disintegration of the nervous tissue, either from maceration in serum, by hemorrhagic extravasation, putrefaction, or mechanical violence, and it differs from the preceding in not causing any symptoms, being, in fact, a *post-mortem* appearance. It is, however, impossible to distinguish the two with any certainty by the unaided eye. As respects colour, the fawn-coloured is most commonly inflammatory; the red usually depends on congestion or extravasated blood; the yellow on the colouring matter of the blood; the fawn or gray-coloured on the presence of brown exudation-corpuscles. Maceration in serum causes a white softening, but the presence or infiltration of pus has never been detected as a cause of this morbid change. As respects the symptoms, contraction in one or more limbs is a common symptom of idiopathic softening of the brain. Inflammation of the central parts of the brain generally produce well-marked lesions of sensation and motion, whilst in inflammation of the peripheral portions, lesions of intelligence are commonly exhibited.

